# An Extremely Rare Case of Metastatic Merkel Carcinoma of the Liver

**DOI:** 10.7759/cureus.19659

**Published:** 2021-11-17

**Authors:** Mathew Thomas, Amrendra Mandal

**Affiliations:** 1 Internal Medicine, State University of New York Upstate Medical University, Syracuse, USA; 2 Internal Medicine/Gastroenterology, State University of New York Upstate Medical University, Syracuse, USA

**Keywords:** nivolumab, liver, neuroendocrine, cutaneous t cell lymphoma, merkel cell carcinoma

## Abstract

Merkel cell carcinoma (MCC) is a rare but highly aggressive skin cancer with neuroendocrine features. The important risk factors in the development of MCC include immunocompromised state, advanced age, and White skin complexion. The pathogenesis is associated with either the presence of Merkel cell polyomavirus or chronic exposure to ultraviolet radiation. MCC usually occurs in the sun-exposed areas of the skin and has the potential for regional and distant metastasis.

Only 8% of the cases of MCC present with distant metastatic disease with liver, lungs, bone, and brain being commonly involved. Here, we report such an extremely rare case of a 78-year-old gentleman with a history of cutaneous T-cell lymphoma, who presented to the hospital with abnormally elevated liver enzymes, which on further evaluation was detected to have metastatic MCC of the liver.

## Introduction

Merkel cells are found as single cells within the basal layer of the epidermis. Merkel cell carcinoma (MCC) arises in the dermis and frequently extends into the subcutaneous fat. It is an extremely rare cancer with an approximate incidence of 2,500 cases per year in the United States [1]. MCC has a propensity for regional and distant metastasis and is associated with a high mortality rate with the five-year overall survival rate of 14%-51% depending on the extent of the disease at presentation [2].

## Case presentation

Our patient is a 78-year-old male with a past medical history of cutaneous T-cell lymphoma/mycosis fungoides (on regular outpatient extracorporeal photopheresis), type II diabetes mellitus, atrial flutter on Xarelto, and sick sinus syndrome on dual-chamber pacemaker, presented to the hospital with right upper quadrant abdominal pain. The patient was a former smoker and denied any alcohol use.

In the emergency department, he was hemodynamically stable. Laboratory workup was significant for abnormally elevated liver function tests including aspartate aminotransferase/alanine aminotransferase (AST/ALT) of 204/188 U/L, alkaline phosphatase (ALP) of 550 U/L, and total bilirubin of 2.5 mg/dL. Ultrasound of the abdomen was negative for any focal liver or gallbladder lesions. There was no evidence of intrahepatic or extrahepatic biliary duct dilation. Hepatobiliary iminodiacetic acid (HIDA) scan was normal, and hence cholecystitis was ruled out. CT abdomen and pelvis and CT angiography of the chest were negative for acute pathology. As the patient had a pacemaker, magnetic resonance cholangiopancreatography (MRCP) could not be performed. Further laboratory evaluation for elevated liver enzymes, including viral hepatitis panel, thyroid-stimulating hormone (TSH), iron panel, antinuclear antibody (ANA), anti-mitochondrial antibody, alpha-1-antitrypsin antibody, anti-smooth muscle antibody, and ceruloplasmin was negative.

**Figure 1:**
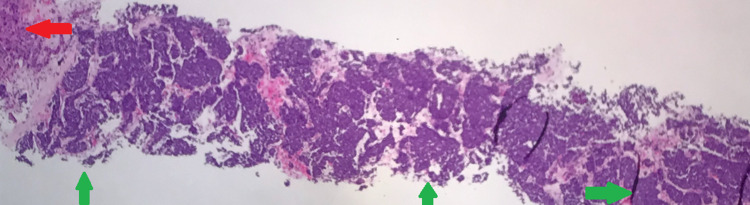
Normal liver parenchyma (red arrow) replaced by a high-grade tumor (green arrow) with high nuclear to cytoplasmic ratio

**Figure 2:**
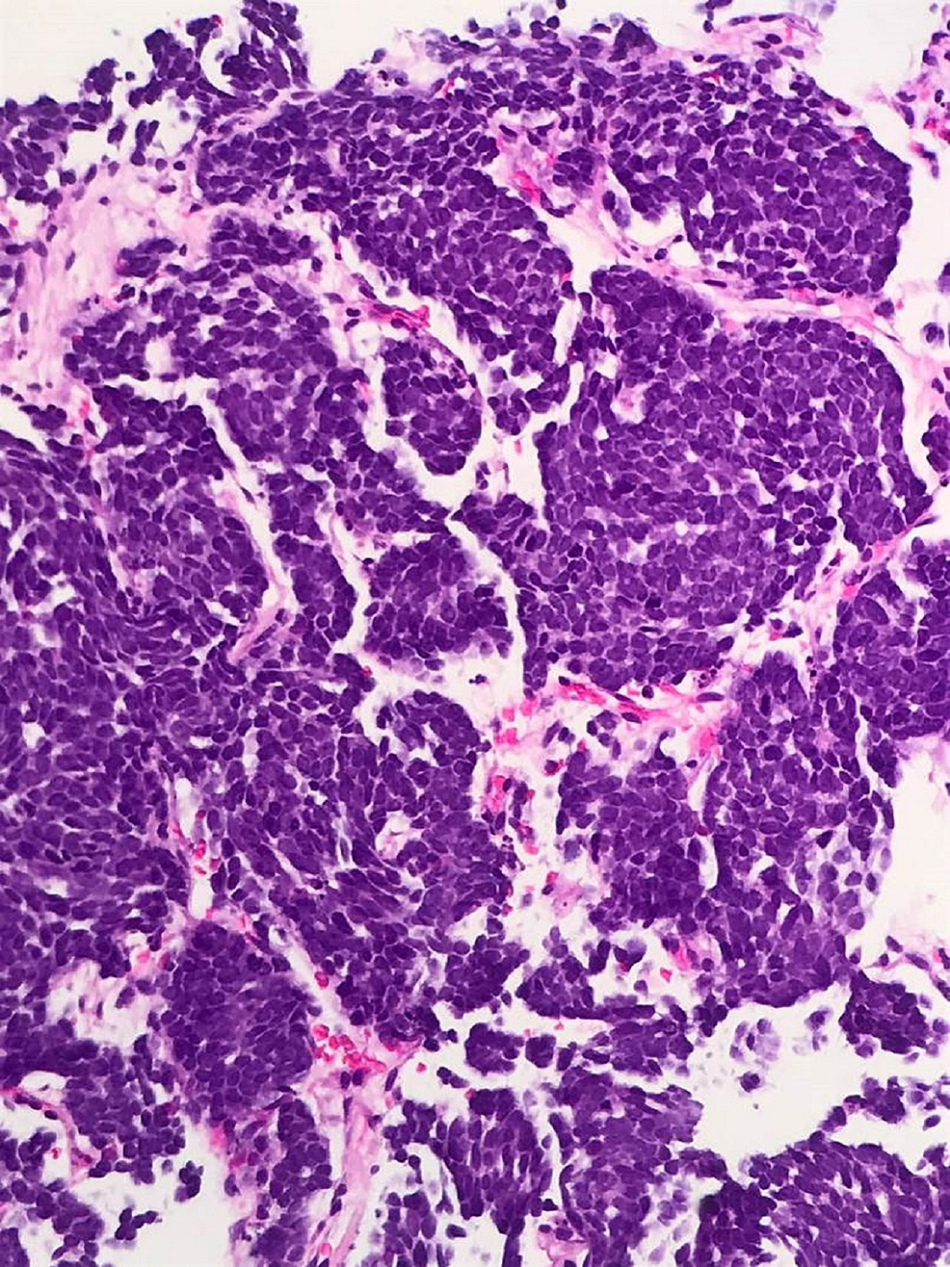
High-grade tumor with high nuclear to cytoplasmic ratio

**Figure 3:**
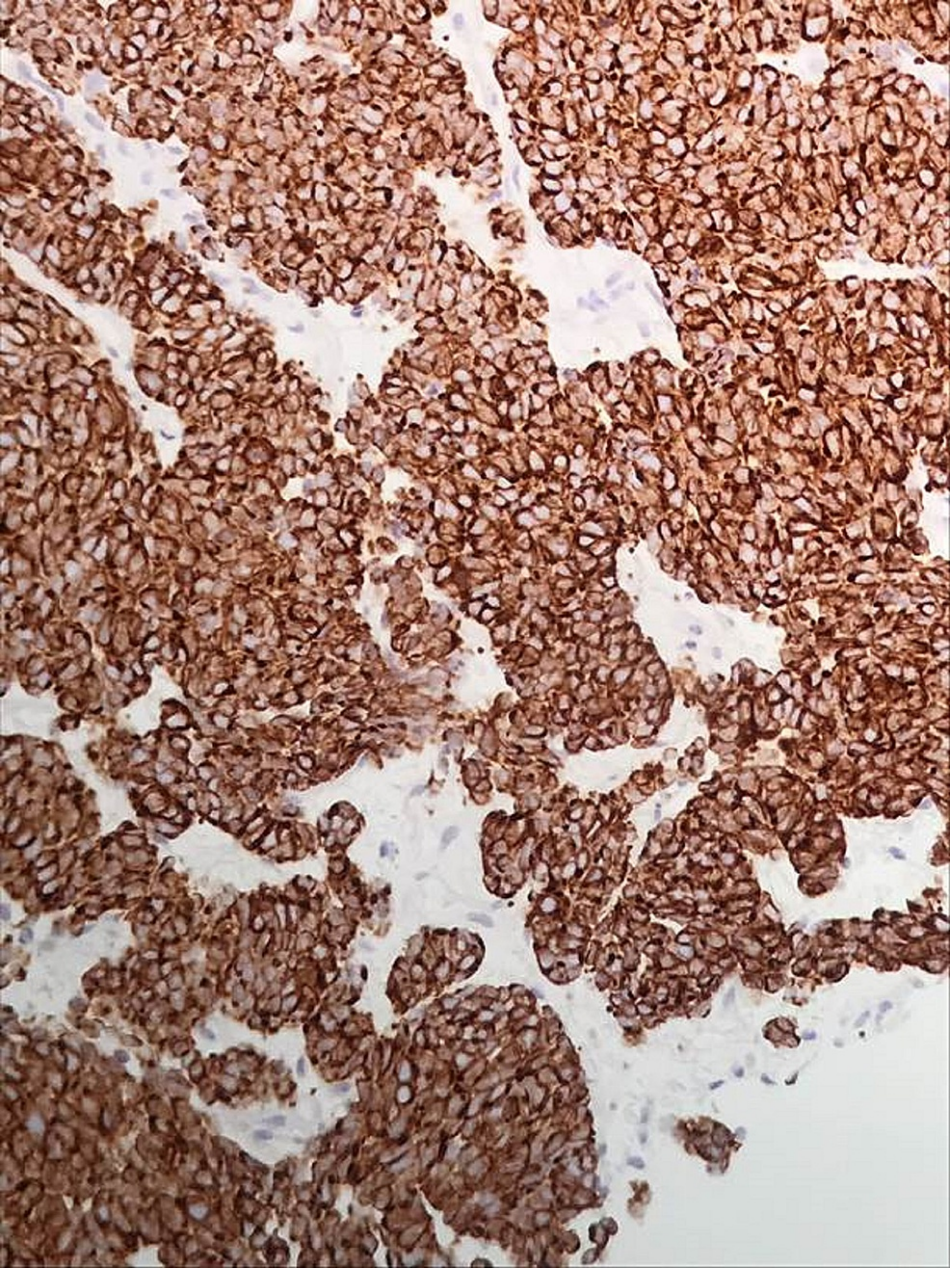
Tumor cells demonstrating positive immunohistochemical staining for cytokeratin AE1/AE3

**Figure 4:**
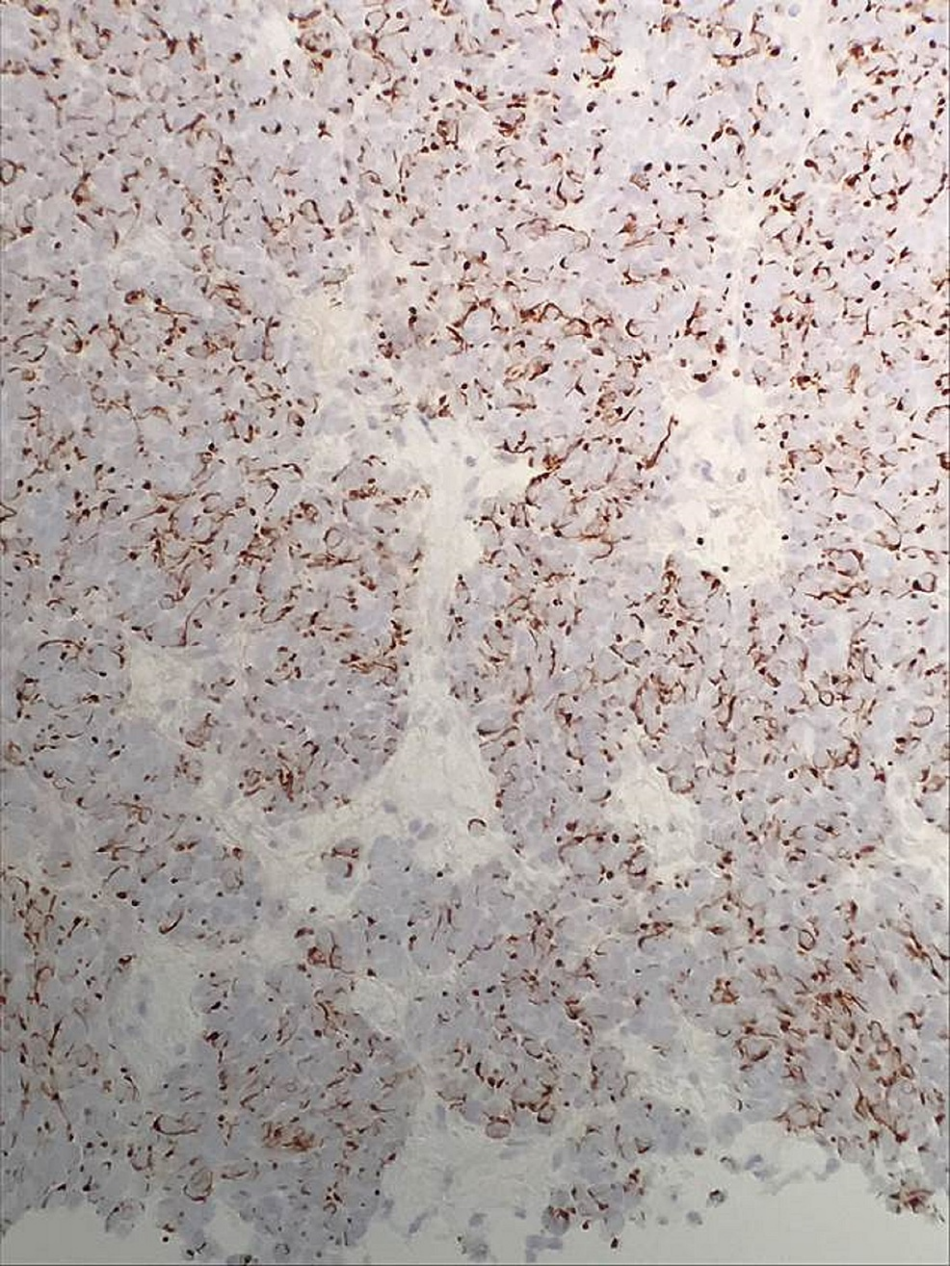
Tumor cells demonstrating positive immunohistochemical staining for cytokeratin 20 (CK20)

**Figure 5:**
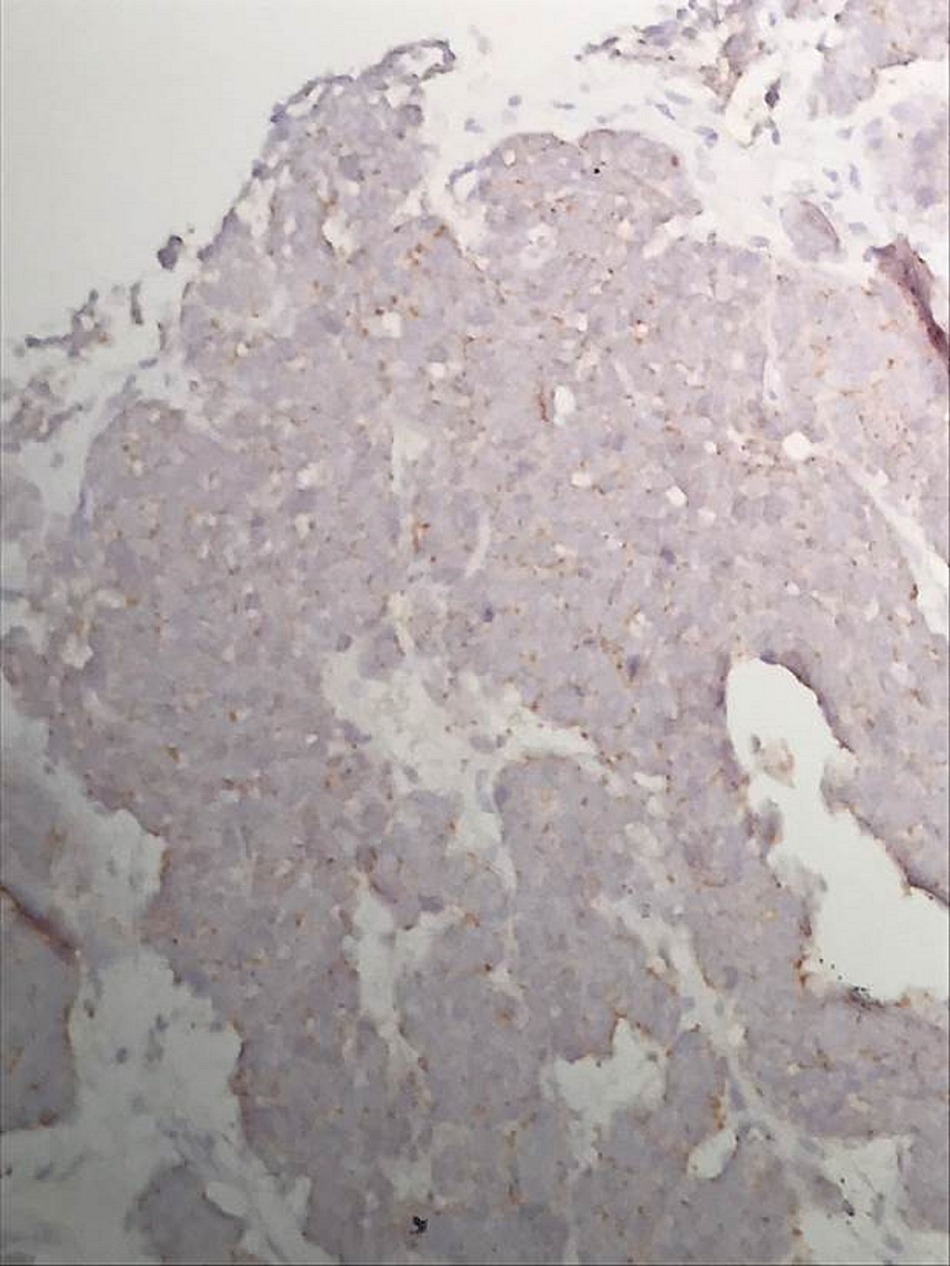
Tumor cells demonstrating positive immunohistochemical staining for synaptophysin

**Figure 6:**
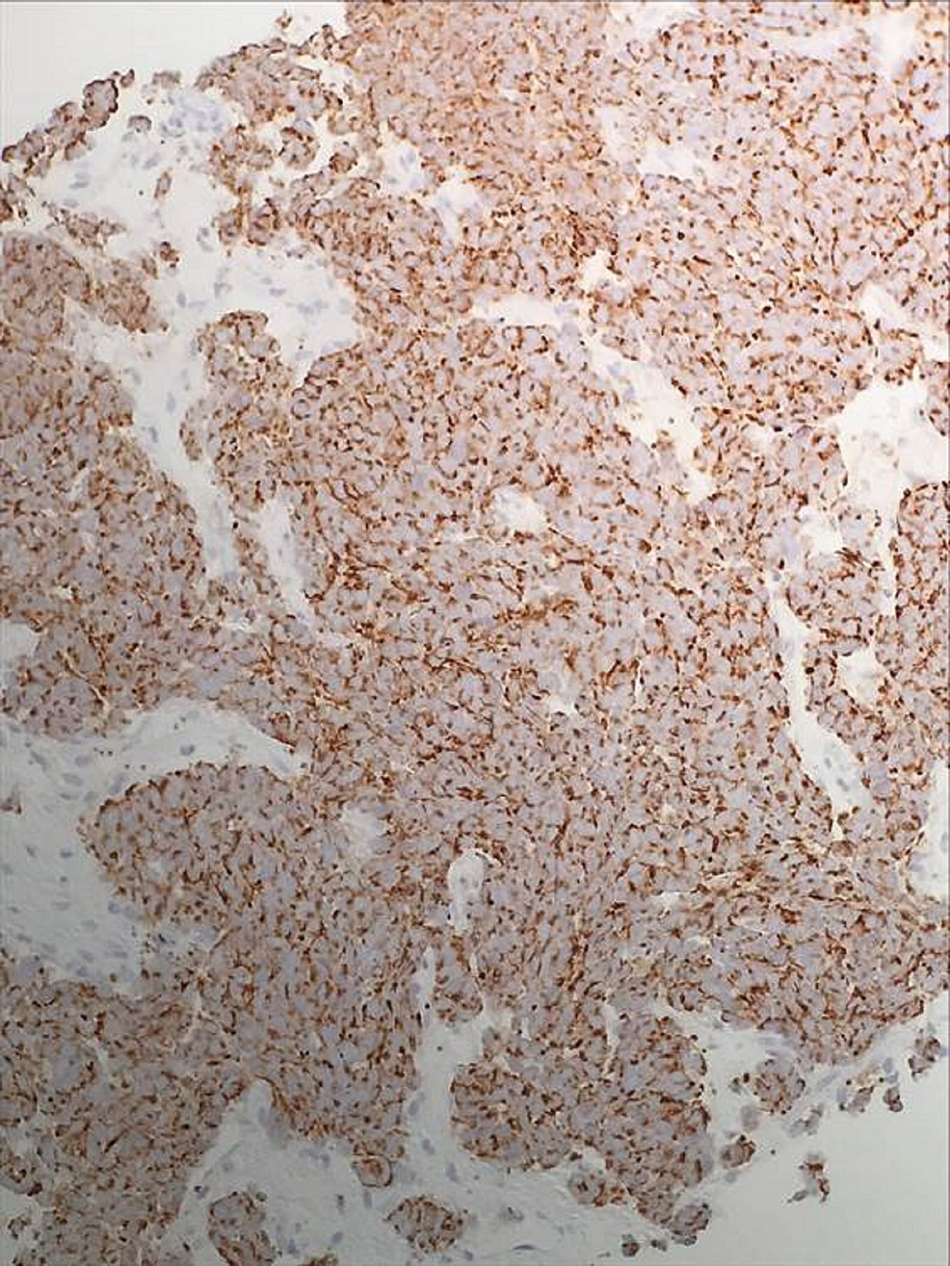
Tumor cells demonstrating positive immunohistochemical staining for chromogranin

Given that the patient has a history of cutaneous T-cell lymphoma, the important differential diagnosis included leukemic infiltration of the liver and adverse reaction to the prior chemotherapy. However, the patient received only a short course of the chemotherapeutic regimen mogamulizumab (due to insurance issues), and hence it was unlikely to cause this current clinical picture. Subsequently, a percutaneous liver biopsy was performed to confirm the diagnosis, which showed replacement of the normal liver parenchymal cells by high-grade tumor cells with a high nuclear-cytoplasmic ratio (Figures 1-2). The tumor cells showed positive immunohistochemical staining for cytokeratin AE1/AE3, cytokeratin 20 (CK20), synaptophysin, chromogranin, and negative for CK7, caudal type homeobox transcription factor 2 (CDX-2), and thyroid transcription factor 1 (TTF-1) (Figures 3-6). All these features were suggestive of metastatic Merkel cell carcinoma. There was no evidence of leukemic infiltrates. As the patient had no evidence of MCC involvement of the skin, he was diagnosed with metastatic MCC of the liver of unknown primary. 

 

 

 

 

 

Hematology/Oncology and Dermatology was consulted. Considering the medical comorbidities, the patient and family opted for comfort care measures and were discharged home. 

## Discussion

Merkel cell carcinoma (MCC) is a highly aggressive cutaneous neuroendocrine carcinoma [2]. Merkel cells are present in the basal layer of the epidermis and serve as the mechanoreceptors for gentle touch [3]. The important risk factors associated with MCC include light skin complexion, advanced age, male sex, and immunocompromised state [3]. Patients with MCC frequently have a history of other ultra-violet (UV) radiation-associated skin cancer (basal cell carcinoma or squamous cell carcinoma) [4]. A history of melanoma is associated with a threefold increased risk of MCC [5]. MCC is more frequent in patients with leukemia, lymphoma, and other immunosuppressed conditions including HIV and organ transplantation [6,7]. The increased incidence of MCC in the White population compared to Black, Asian, and Hispanic population explains the protective effect of skin pigmentation against MCC [3].

The etiopathogenesis of MCC involves the clonal integration of the Merkel cell polyomavirus (MCPyV) DNA into the MCC cell genome or somatic mutation from chronic UV radiation mediated DNA damage [3]. UV radiation can also induce the expression of inflammatory mediators and alter the antigen-presenting dendritic cells, thereby resulting in a series of events that modulate the immune sensitivity [3]. UV radiation can cause local immunosuppression which could also play a role in viral carcinogenesis [8]. Despite the significant advances in molecular biology, the cellular origin of MCC remains unclear. MCC is hypothesized to be originating from Merkel cell precursors (derived from epidermal stem cells or hair follicle stem cells), pre-B cells, pro-B cells, or dermal fibroblasts [9]. As a normal Merkel cell is terminally differentiated and does not undergo cell division, they are unlikely to be the cell of origin for MCC [3].

Most cases of MCC present as a rapidly growing solitary cutaneous or subcutaneous nodule on the sun-exposed areas of the skin [10]. Routine studies with hematoxylin/eosin and immunohistochemical stains are required to diagnose MCC. On immunohistochemistry, Merkel cells show features of both epithelial and neuroendocrine cells [11]. The important epithelial markers include AE1/AE3, CAM 5.2, pan-cytokeratin, epithelial membrane antigen, and Ber-EP4 [12]. The significant neuroendocrine markers include chromogranin, synaptophysin, calcitonin, vasoactive intestinal peptide, and somatostatin receptor [12].

About 65% of patients with MCC present with localized disease, 26% with regional lymph node involvement, and 8% with distant metastatic disease. Wide excision of the primary tumor is the standard of care for localized disease [13]. For patients who are not surgical candidates, radiation is the alternative treatment option [3]. Wide local excision of the primary lesion with sentinel lymph node biopsy is the treatment of choice for loco-regional disease. If the lymph node is positive for cancer involvement, lymph node surgery with or without radiation therapy is preferred [14]. Before the introduction of immunotherapy, chemotherapeutic regimens including platinum-based regimens, etoposide, taxanes, and anthracyclines, either alone or in combinations were the standard of care for metastatic MCC, not amenable to surgery or radiation [3,15]. The extent of disease at presentation and regional lymph node involvement is considered as the most important predictive factor for survival of MCC. The five-year overall survival (OS) ranges from 50.6% for patients with local disease to 13.5% for patients with distant metastasis [2].

Following the introduction of immunotherapy, several studies have demonstrated that targeting the immune checkpoint pathway could be an effective approach in MCC. A phase II study conducted by Nghiem et al. demonstrated that first-line therapy with pembrolizumab in patients with advanced Merkel cell carcinoma was associated with an objective response rate of 56% [16]. A phase II multicenter study conducted by Kaufman et al. investigated the efficacy of avelumab in chemotherapy-refractory advanced-stage MCC. It demonstrated that 82% of the responded patients maintained their initial response at a median follow-up of 10.4 months. This led to the approval of avelumab by the FDA in March 2017 in the second-line setting of metastatic MCC [17]. The phase I/II CheckMate 358 study conducted by Topalian et al. demonstrated that the use of nivolumab in the neo-adjuvant setting is generally tolerable, induced pathological complete response (pCR) and radiographic tumor regressions in approximately 50% of the patients [18].

As there are not much data available on the management of MCC with liver metastasis, the treatment should be individually tailored following multidisciplinary discussion. The utilization of clinical trials for novel treatment options may be considered. 

## Conclusions

Merkel cell carcinoma (MCC) is an aggressive skin cancer with the propensity for regional and distant metastasis, including liver, lung, and brain, occurring in only 8% of the cases. Metastatic MCC can occasionally present with an unknown primary skin tumor. Our patient was diagnosed with metastatic MCC to the liver with unknown primary. Immunocompromised state (from other malignancy, HIV, organ transplantation), advanced age, and White skin complexion are considered to be the important risk factors for MCC. The five-year overall survival rate for distant metastatic disease is only 14%; hence, prompt initiation of treatment is of utmost importance.
